# A hamster model for Marburg virus infection accurately recapitulates Marburg hemorrhagic fever

**DOI:** 10.1038/srep39214

**Published:** 2016-12-15

**Authors:** Andrea Marzi, Logan Banadyga, Elaine Haddock, Tina Thomas, Kui Shen, Eva J. Horne, Dana P. Scott, Heinz Feldmann, Hideki Ebihara

**Affiliations:** 1 Laboratory of Virology, Division of Intramural Research, National Institute of Allergy and Infectious Diseases, National Institutes of Health, Hamilton, MT 59840, USA; 2 Bioinformatics and Computational Biosciences Branch, National Institute of Allergy and Infectious Diseases, National Institutes of Health, Bethesda, MD 20892, USA; 3 Rocky Mountain Veterinary Branch, Division of Intramural Research, National Institute of Allergy and Infectious Diseases, National Institutes of Health, Hamilton, MT 59840, USA

## Abstract

Marburg virus (MARV), a close relative of Ebola virus, is the causative agent of a severe human disease known as Marburg hemorrhagic fever (MHF). No licensed vaccine or therapeutic exists to treat MHF, and MARV is therefore classified as a Tier 1 select agent and a category A bioterrorism agent. In order to develop countermeasures against this severe disease, animal models that accurately recapitulate human disease are required. Here we describe the development of a novel, uniformly lethal Syrian golden hamster model of MHF using a hamster-adapted MARV variant Angola. Remarkably, this model displayed almost all of the clinical features of MHF seen in humans and non-human primates, including coagulation abnormalities, hemorrhagic manifestations, petechial rash, and a severely dysregulated immune response. This MHF hamster model represents a powerful tool for further dissecting MARV pathogenesis and accelerating the development of effective medical countermeasures against human MHF.

Marburg virus (MARV), a non-segmented, negative sense RNA virus belonging to the family *Filoviridae*, has been responsible for causing sporadic outbreaks of hemorrhagic fever throughout central Africa since its discovery in 1967^1^. The largest and deadliest of these outbreaks occurred in 2004–2005 in the Uíge province of Angola, where 252 cases of Marburg hemorrhagic fever (MHF) were reported and 227 people died^2^. With a case fatality rate of ~90%, the Angolan outbreak of MARV was one of the deadliest filovirus outbreaks on record, rivaling or exceeding the severity of outbreaks caused by the related Ebola virus (EBOV)^1^,^2^. MARV has also been inadvertently imported to other nations on numerous occasions, including two recent cases in the Netherlands and United States, one of which resulted in a fatality^1^,^3^,^4^.

Along with the distinct Ravn virus, MARV belongs to the genus *Marburgvirus* and comprises several different variants, including Musoke, Ci67, Popp, Ozolin, and Angola^1^. Of these, MARV-Angola seems to be the most virulent, as indicated by pathogenesis experiments in non-human primates (NHPs) and guinea pigs, as well as the extremely high case fatality rate in the Angolan outbreak^2^,^5^,^6^,^7^. Moreover, several studies have reported the successful use of aerosolized MARV, including variant Angola, to lethally infect NHPs^8^,^9^,^10^,^11^. Accordingly, MARV is considered a significant public health threat and is classified as a Category A Bioterrorism Agent by the Centers for Disease Control and a Tier 1 Select Agent by the United States Department of Health and Human Services. Moreover, the World Health Organization has recently designated MARV a priority pathogen needing urgent research attention^12^.

The 2013–2016 EBOV epidemic in West Africa highlighted the significant threat that filovirus outbreaks pose to international public health, yet despite the potential for MARV to cause serious outbreaks^1^,^3^,^4^, there are still no medically licensed vaccines or therapeutics to treat MHF. The development of such countermeasures requires relevant and well-characterized animal models that closely recapitulate the disease observed in humans. NHPs, particularly macaques, are considered the “gold-standard” model for MHF since they closely reflect human disease^13^,^14^. However, due to ethical and practical concerns, the use of NHPs is typically limited to the final evaluation of preclinical vaccine and therapeutic trials. Rodents therefore represent an important model system for initial evaluation of preventative and post-exposure countermeasures, and while several rodent MHF models have been developed, none of them recapitulates the disease as completely as it is observed in humans and NHPs^14^. In particular, the mouse models demonstrate only limited or inconsistent coagulation abnormalities, and neither the mouse nor the guinea pig models exhibit the full range of hemorrhagic manifestations, including the characteristic petechial rash^7^,^15^,^16^.

We used Syrian golden hamsters, which are widely used as animal models for infectious diseases^17^,^18^, to produce a rodent model that more precisely reflects MARV infection in humans and NHPs. Using hamster-adapted (HA) MARV-Angola, we performed a detailed pathological analysis of infection over time. Not only did HA-MARV infection of hamsters reproduce nearly all of the clinical features of MHF observed in humans and NHPs, including severe hematological and coagulation abnormalities, but it also reproduced hemorrhagic manifestations and the characteristic rash, which has never before been observed in any other filovirus rodent model. Moreover, hamsters infected with HA-MARV demonstrated an early and dysregulated innate immune response, which we suspect is a critical contributor to pathogenesis. Overall, this hamster model of MHF represents the closest approximation of human MHF outside of NHPs, and it will be an invaluable tool in the study of MARV pathogenesis and the development of viral countermeasures.

## Results

### Establishment of a Lethal Hamster Model Recapitulating Marburg Hemorrhagic Fever

To develop a lethal model for MARV infection in Syrian golden hamsters (*Mesocricetus auratus*), wild-type (WT) MARV variant Angola was serially passaged three times in Hartley guinea pigs (*Cavia porcellus*) until uniform lethality was achieved (data not shown). The resulting guinea pig-adapted MARV (GPA-MARV) isolate was then serially passaged five times in hamsters to obtain hamster-adapted MARV (HA-MARV). Sequence analysis of HA-MARV revealed a total of 61 unique mutations relative to the parental WT-MARV, although only three of these mutations were non-synonymous (Table 1). A single amino acid mutation was observed in VP35, and two were observed in VP40. The vast majority of the changes occurred in the VP35 5′ non-coding region, which contained 54 U to C mutations.

**Figure 1:**
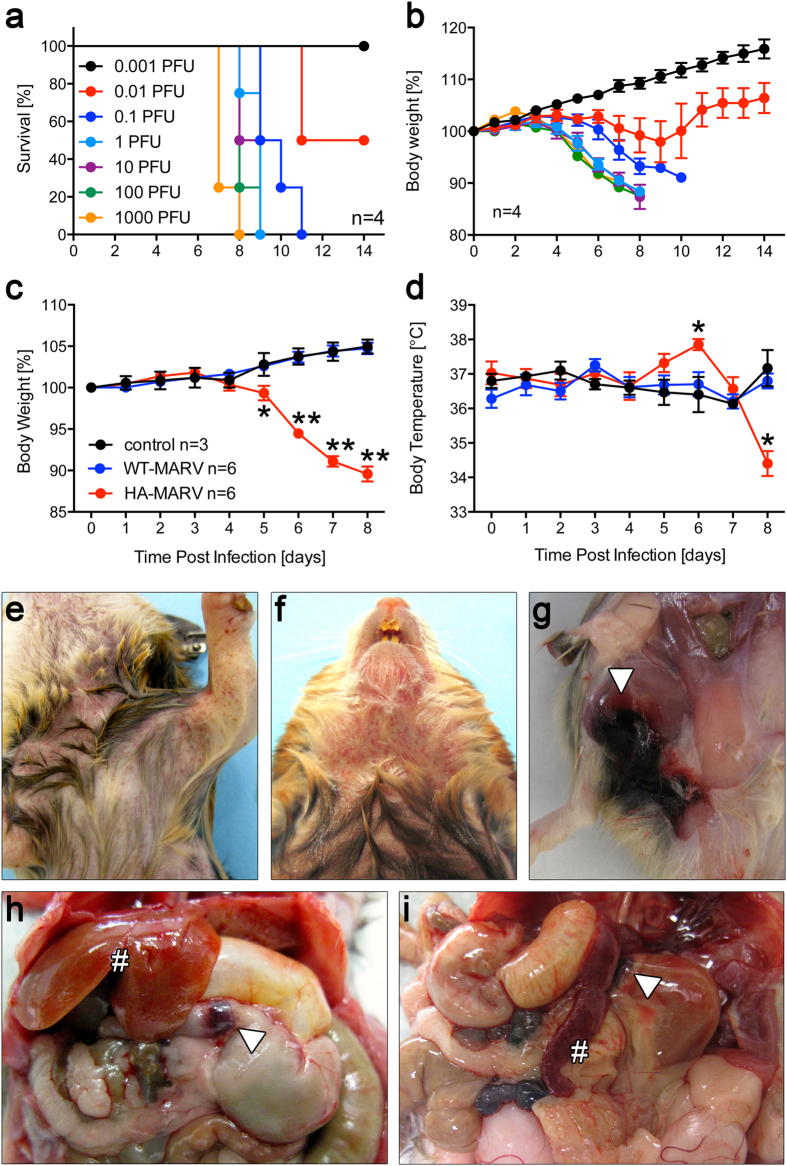
Establishment of a lethal hamster model for Marburg virus. (**a,b**) Seven groups of 4 hamsters each were inoculated intraperitoneally with 0.001 plaque-forming units (PFU) to 1000 PFU HA-MARV. (**a**) Survival of the animals is presented as a Kaplan-Meier survival curve. (**b**) Percentage weight loss expressed as the mean ± SEM. (**c**–**i**) Eight groups of 6 hamsters were inoculated intraperitoneally with 100 LD_50_ (1 PFU) HA-MARV and 8 groups of 6 hamsters were inoculated with an equivalent dose of WT-MARV. Six hamsters from each group were sacrificed and samples collected each day post-infection, up to day 8. Six control hamsters were mock infected; 3 were sacrificed on day 1 and 3 on day 8. (**c**) Percentage weight loss over the course of the experiment of all animals sacrificed on day 8, expressed as the mean ± SEM. (**d**) Body temperatures over the course of the experiment of all animals sacrificed on day 8, expressed as the mean ± SEM. Two of the six animals were found dead on day 8, so n = 4 at this timepoint only. (**e,f**) A disseminated maculopapular/petechial rash was visible on hamsters 7 (**e**) and 8 (**f**) days post-infection. (**g–i**) Gross lesions revealed by necropsy included a large inguinal subcutaneous hemorrhage (arrowhead) after 7 days post-infection (**g**) swollen, soft, pale livers (#) and gastro-duodenal hemorrhaging (arrowhead) 7 days post-infection (**h**) and swollen, soft, pale spleens (#) and hemorrhaging of the adrenal cortex (arrowhead) 8 days post-infection (**i**). *p*-values ≤ 0.05 are indicated by a single asterisk (*) and *p*-values ≤ 0.001 are indicated by two asterisks (**), compared to WT-MARV.

Intraperitoneal inoculation of hamsters with 0.1 to 1000 PFU HA-MARV resulted in significant weight loss and uniform lethality, with hamsters succumbing to disease between 7 and 11 days post-infection (Fig. 1a,b). None of the hamsters inoculated with 0.001 PFU lost weight or succumbed to infection, whereas half of the hamsters inoculated with 0.01 PFU died (Fig. 1a,b). Accordingly, the dose at which 50% of animals succumb to infection (LD_50_) was calculated to be 0.01 PFU.

To characterize the pathogenesis of HA-MARV, hamsters were intraperitoneally inoculated with either a uniformly lethal dose of 100 LD_50_ (1 PFU) HA-MARV or an equivalent dose of parental WT-MARV variant Angola. Six additional hamsters were mock infected. Hamsters infected with HA-MARV showed a decrease in activity and began to lose a significant amount of weight beginning around day 5 post-infection and continuing until day 8 (Fig. 1c). Moreover, HA-MARV-infected hamsters exhibited a significant spike in body temperature at day 6 post-infection followed by a dramatic drop in temperature during the terminal stage of the disease (Fig. 1d). Conversely, animals that were mock infected or infected with WT-MARV did not exhibit signs of disease, including changes in body temperature, and gained weight throughout the study, suggesting that WT-MARV was incapable of producing disease in hamsters (Fig. 1c,d).

Strikingly, we observed several hemorrhagic manifestations in hamsters infected with HA-MARV, consistent with what has been observed in humans and NHPs^19^,^20^,^21^. Most notably, by day 7 post-infection all HA-MARV-infected hamsters developed a maculopapular rash along with petechiae visible on the face, chin, chest, abdomen, and limbs that persisted until the humane endpoint was reached (Fig. 1e,f). Additional gross lesions included an inguinal subcutaneous hemorrhage (1/6 animals on day 7; Fig. 1g), along with hemorrhaging at the gastro-duodenal junction (3/6 and 3/4 animals on days 7 and 8, respectively; Fig. 1h), at the adrenal cortex (1/6 and 4/4 animals on days 7 and 8, respectively; Fig. 1i), at the small intestine (2/6 and 4/4 animals on days 7 and 8, respectively; data not shown), and at the cervical lymph nodes (1/6 and 3/4 animals on days 7 and 8, respectively; data not shown). Hemorrhaging was also observed sporadically in the footpads (ecchymosis) and joints as well as the kidneys (data not shown).

### HA-MARV Induces Coagulation and Hematologic Abnormalities

**Figure 2:**
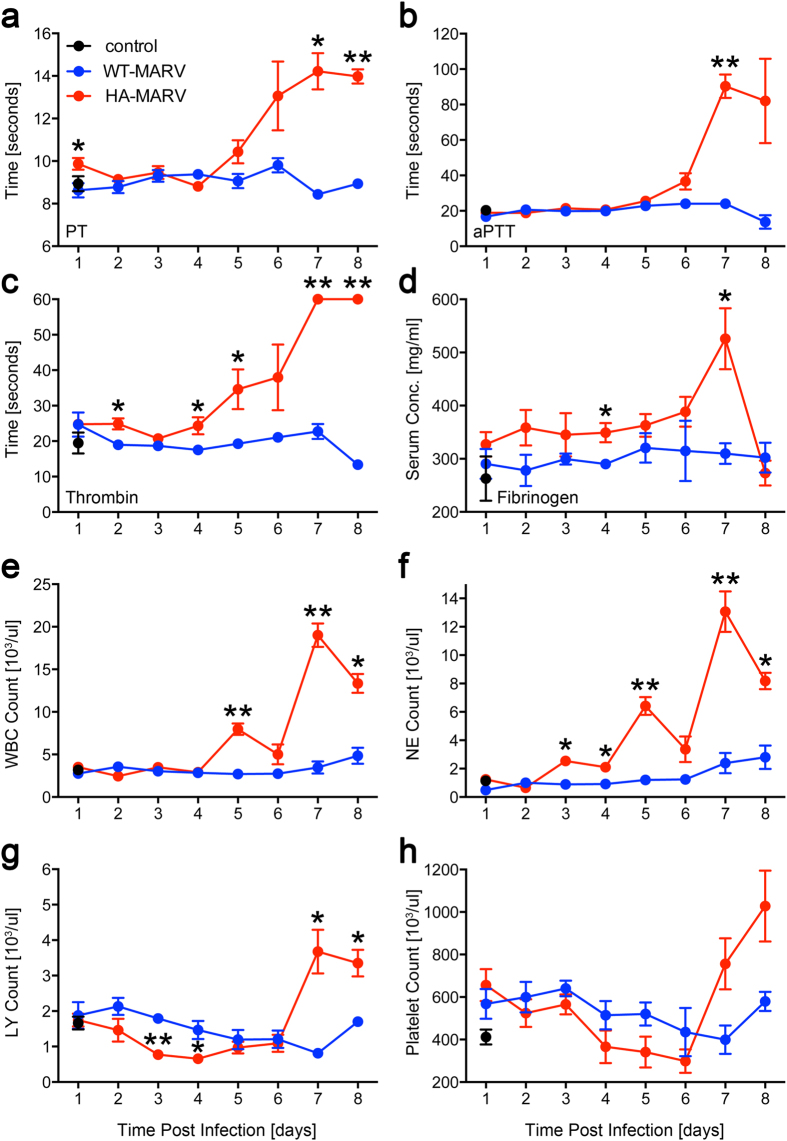
HA-MARV induces hematologic and coagulation abnormalities. (**a–h**) Blood samples were collected from sacrificed animals each day post-infection with HA- or WT-MARV and analyzed for prothrombin time (PT) (**a**) activated partial thromboplastin time (aPTT) (**b**), thrombin time (**c**) fibrinogen serum concentration (**d**) white blood cell (WBC) count (**e**) total neutrophil (NE) count (**f**) total lymphocyte (LY) count (**g**) and platelet count (**h**). All values are expressed as the mean ± SEM. n = 3–6 for the infected groups, n = 3 for the control group. *p*-values ≤ 0.05 are indicated by a single asterisk (*) and *p*-values ≤ 0.001 are indicated by two asterisks (**), compared to WT-MARV.

Analysis of coagulation parameters in hamsters infected with HA-MARV indicated severe coagulopathy, consistent with what has been observed in NHPs and humans^19^,^20^,^21^,^22^. Prothrombin (PT), activated partial thromboplastin (aPTT), and thrombin times in HA-MARV-infected hamsters were all significantly elevated late during the course of infection, indicating delays in blood clot formation (Fig. 2a–c). Fibrinogen levels remained slightly elevated throughout infection and peaked on day 7 before dropping dramatically on day 8 (Fig. 2d). Conversely, animals infected with WT-MARV displayed no significant change in these values, showing parameters similar to what was observed in control animals (Fig. 2a–d).

Hematological findings in HA-MARV-infected hamsters were similar to those observed in other models of filovirus infection^14^,^20^,^21^. Late-stage leukocytosis in animals infected with HA-MARV was indicated by elevated white blood cell counts, which rose to levels significantly higher than in WT-MARV-infected hamsters at day 5 post-infection and peaked at day 7 (Fig. 2e). Neutrophilia was the most likely cause of leukocytosis since significantly higher numbers of neutrophils were detected throughout the course of disease, peaking on day 7 post-infection (Fig. 2f). Lymphopenia was observed in animals infected with HA-MARV on days 3 and 4 post-infection, followed by an increase in lymphocyte numbers on days 7 and 8 (Fig. 2g). Although platelet levels were lower in HA-MARV-infected animals from days 2 to 6 post-infection, the difference was not statistically significant (Fig. 2h). Interestingly, however, platelet levels did spike in HA-MARV-infected animals on days 7 and 8, similar to what has been observed previously in cynomolgus macaques infected with MARV variant Ci67^23^. Overall, these disturbances in coagulation and hematological factors are consistent with disseminated intravascular coagulation (DIC), which is typical of hemorrhagic fevers and is likely to have contributed to the hemorrhagic manifestations observed in this model^20^.

### HA-MARV Replicates More Robustly than WT-MARV

**Figure 3:**
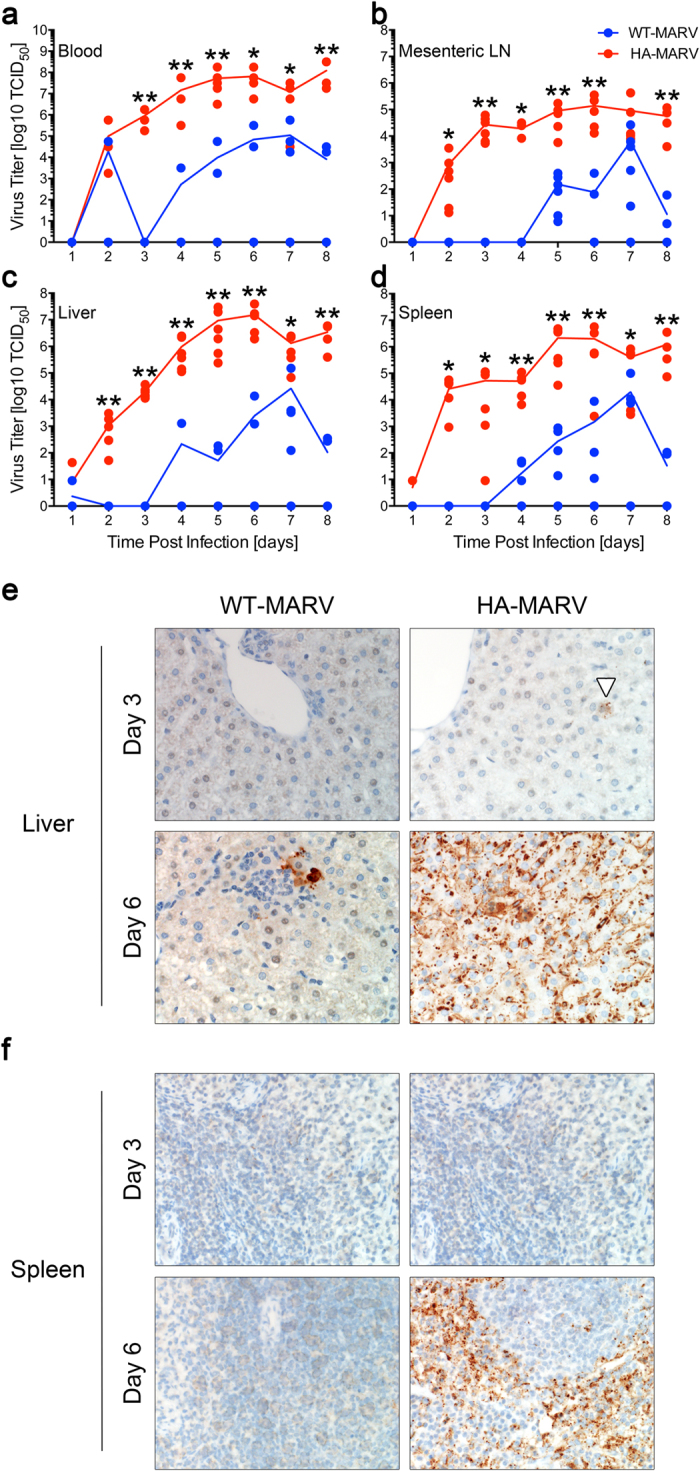
HA-MARV replicates robustly in hamsters. (**a–d**) Virus titers, expressed as tissue culture infectious dose 50% (TCID_50_) on a log10 scale, were calculated from the blood (**a**) the mesenteric lymph nodes (**b**) the livers (**c**) and the spleens (**d**). Mean values for each time point are indicated by the line graph, and individual values for each hamster are indicated by blue (WT-MARV) or red (HA-MARV) dots. n = 3–6. *p*-values ≤ 0.05 are indicated by a single asterisk (*) and *p*-values ≤ 0.001 are indicated by two asterisks (**) compared to WT-MARV. (**e,f**) Immunohistochemistry (IHC) to detect MARV antigen was performed on liver (**e**) and spleen (**f**) samples collected 3 or 6 days post-infection with either WT- or HA-MARV. A focus of virus antigen in the liver of a HA-MARV-infected hamster at day 3 is indicated by a white arrowhead. Note that the WT-MARV-infected hamster randomly selected for IHC analysis exhibited no detectable virus titer in the spleen and a very low titer in the liver, which correlates with the absence of antigen detected by IHC in the spleen and the small amount detected in the liver.

HA-MARV replication in hamsters was systemic and robust, reaching titers that peaked around 10^8^ TCID_50_/ml in the blood, 10^7^ TCID_50_/ml in the liver, 10^6^ TCID_50_/ml in the spleen, and 10^5^ TCID_50_/ml in the mesenteric lymph nodes (Fig. 3a–d). High titers of infectious virus were also detected in numerous additional tissues at days 7 and 8 post-infection (Supplementary Fig. 1). Replication of WT-MARV was considerably poorer, with no more than two-thirds of the animals exhibiting detectable virus and with titers up to 3 logs lower than HA-MARV (Fig. 3a–d).

The robust replication of HA-MARV was confirmed by immunohistochemistry of liver and spleen samples (Fig. 3e,f). Small numbers of cells with morphology consistent with endothelial cells, hepatocytes, and Kupffer cells were positive for MARV antigen by day 3 post-infection with HA-MARV, and the majority of these cells were positive by day 6. Only a small number of hepatocytes were positive for viral antigen on day 6 post-infection with WT-MARV, and no cells were positive after this time point (Fig. 3e). Similarly, viral antigen was detected in small numbers of mononuclear cells in the red pulp of the spleen at 3 days post-infection with HA-MARV, and by day 6 post-infection viral antigen was concentrated in cells at the marginal zone and throughout the entirety of the red pulp (Fig. 3f). In addition, small numbers of mononuclear cells were positive for MARV antigen in the lymphoid follicles of the spleen (Fig. 3f). No MARV antigen was detected at any time point in the spleens of animals infected with WT-MARV (Fig. 3f). Likewise, no antigen was detected in the mock-infected control animals (Supplementary Fig. 2).

### HA-MARV Histopathology is Characteristic of Filovirus Infections

**Figure 4:**
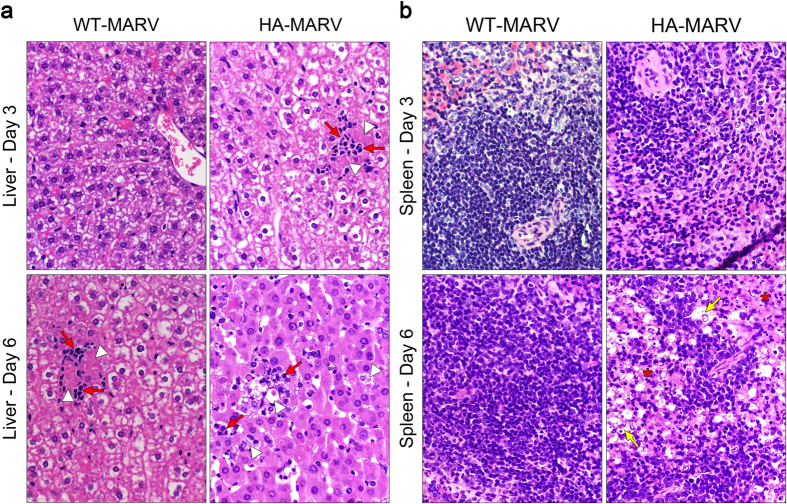
HA-MARV induces pathological changes in the liver and spleen. (**a,b**) Hematoxylin and eosin stained liver (**a**) and spleen (**b**) samples collected 3 or 6 days post-infection with either WT- or HA-MARV. In the liver (**a**) hepatocellular necrosis is indicated by white arrowheads and neutrophilic infiltration is indicated by red arrows. In the spleen (**b**) tingible body macrophages are indicated with yellow arrows and necrosis and loss of lymphocytes is indicated by asterisks.

Histopathological analysis of tissues revealed systemic damage to internal organs, with hemorrhages becoming more severe as infection progressed. As early as day 3 post-infection, hamsters developed acute necrotic hepatitis, with lesions characterized by multifocal aggregates of small numbers of neutrophils (Fig. 4a, red arrows) admixed with individual necrotic hepatocytes (Fig. 4a, white arrowheads). The development of these lesions correlated with virus replication, which was also detectable by day 3 in the liver (Fig. 3e). By day 6, livers of each hamster began to pale, and by days 7 and 8 all livers were enlarged and soft (Fig. 1h and Supplementary Fig. 3). Hepatic lesions became progressively more severe over time, developing into multifocal to coalescing areas of hepatocellular necrosis (Fig. 4a, white arrowheads) with neutrophilic infiltration (Fig. 4a, red arrows) by day 6 (Fig. 4a), when virus replication in the liver was rampant (Fig. 3e). In contrast, hamsters inoculated with WT-MARV developed only minimal to mild hepatic necrosis (Fig. 4a, white arrowheads) with neutrophilic infiltration (Fig. 4a, red arrows) by day 6, and these lesions did not significantly increase in severity at later time points (Supplementary Fig. 4a). Immunohistochemistry analysis of activated caspase-3, a marker of apoptosis induction, revealed a moderate amount of activated caspase-3-positive hepatocytes and Kupffer cells peaking around day 5 in animals infected with HA-MARV, whereas animals infected with WT-MARV exhibited a delayed and suppressed apoptotic response, consistent with the slower replication kinetics of this virus (Supplementary Fig. 5). Notably, the elevation of liver enzymes, such as alkaline phosphatase (ALP), alanine transaminase (ALT), and aspartate transaminase (AST), in the serum is a key hallmark of MHF pathogenesis^19^,^20^,^21^. The extensive damage observed in the livers of animals infected with HA-MARV suggests that the typical elevation in liver enzymes would be observed; however, blood chemistries were not assessed in this study.

Small numbers of mononuclear cells in the splenic red pulp were positive for HA-MARV antigen at day 3 post-infection (Fig. 3f); however, there were no splenic lesions at this time point (Fig. 4b). By day 5, the spleens of hamsters infected with HA-MARV were mildly enlarged, and by day 8 they were up to three times their normal size, in addition to being soft and pale (Fig. 1i). Histologically, splenitis was characterized on day 5 post-infection by infiltration of the red pulp with small to moderate numbers of neutrophils and fewer macrophages, and by day 7 there was also abundant fibrin within the red pulp. Necrosis and loss of lymphocytes (Fig. 4b, asterisks) in the white pulp admixed with small numbers of tingible body macrophages (Fig. 4b, yellow arrows) was observed on day 5, and became more severe over time, involving the majority of the white pulp by day 7 post-infection. This correlated with the increase in viral antigen in the spleen over time (Fig. 3f). Hamsters inoculated with WT-MARV did not develop any obvious lesions in the spleen (Fig. 4b). Histopathologic scores further highlighted the severity of lesions in livers and spleens of HA-MARV-infected hamsters compared to those of WT-MARV-infected hamsters (Supplementary Fig. 4a,b). As in the liver, a moderate amount of activated caspase-3-positive macrophages and lymphocytes were observed peaking around day 5 in the splenic white pulp of HA-MARV-infected animals, whereas the apoptotic response was delayed and suppressed in WT-MARV-infected animals (Supplementary Fig. 5).

Consistent with the gross pathology (Fig. 1e–i), histopathological analysis also revealed necrosis and hemorrhage of the adrenal cortex (Supplementary Figs 4c and 6); necrosis and hemorrhage of the small intestine, with abundant fibrin in the lamina propria (Supplementary Figs 4d and 6); hemorrhage of the duodenum (Supplementary Fig. 6); and lymphoid necrosis of the cervical lymph nodes (Supplementary Fig. 4e). Late in the disease progression, the haired skin demonstrated multifocal intracorneal pustules consisting of moderate numbers of neutrophils admixed with necrotic epithelial cells, and these pustules were adjacent to subcutaneous capillaries lined by endothelial cells that were positive for MARV antigen (Supplementary Fig. 6). These pustules were grossly evident as the maculopapular/petechial rash observed on all animals infected with HA-MARV (Fig. 1e,f).

### HA-MARV Induces an Early and Strong Innate Immune Response

**Figure 5:**
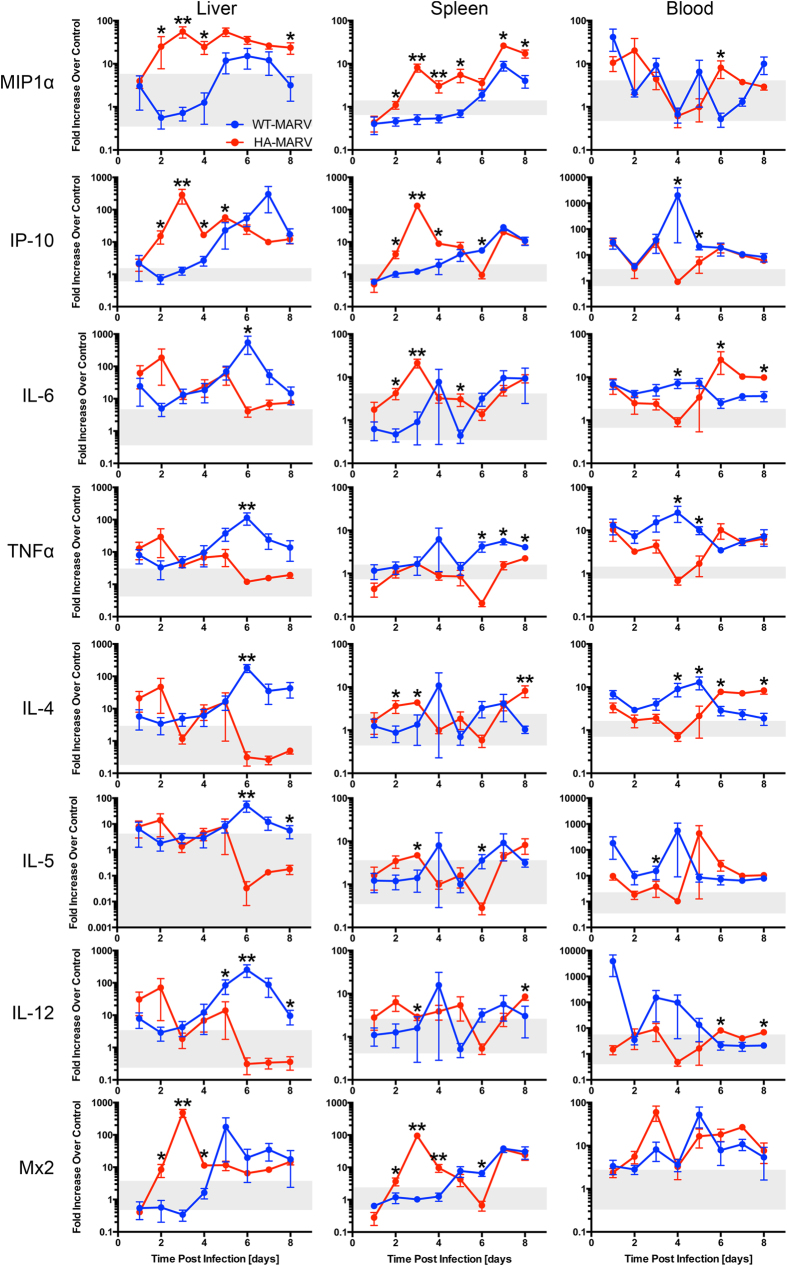
HA-MARV induces an early and strong innate immune response. (**a–c**) Transcript levels for the indicated genes were quantified by RT-qPCR from samples derived from the livers, spleens, and blood of animals infected with WT-MARV (blue lines) or HA-MARV (red lines). Data are expressed as the mean ± SEM of the fold change over uninfected control hamsters on a log10 scale. n = 5–6 for the liver samples and 4–6 for the spleen samples. For the blood samples, n = 3–6, with the exception of HA-MARV day 7, for which n = 1. *p*-values ≤ 0.05 are indicated by a single asterisk (*) and *p*-values ≤ 0.001 are indicated by two asterisks (**) compared to WT-MARV. The grey boxes depict the range of transcript expression observed in uninfected control hamsters, n = 6.

To understand the innate immune response in hamsters infected with MARV, we analyzed the mRNA transcript levels of a variety of cytokines and chemokines from the liver, spleen, and blood. In general, in the liver and spleen, hamsters infected with HA-MARV exhibited a robust response that peaked early during infection, on day 3, and then plateaued or declined in magnitude towards the terminal stage of disease (Fig. 5 and Supplementary Fig. 7a,b, top panels). In contrast, hamsters infected with WT-MARV showed a relatively minor innate immune response early during infection but a significant peak near the end, around days 6 and 7 (Fig. 5 and Supplementary Fig. 7a,b, bottom panels). Notably, in HA-MARV-infected animals, the early innate immune response was primarily driven by the pro-inflammatory chemokines MIP-1α and IP-10, as well as type I interferon responses, as indicated by the Mx2 transcript. All three of these genes, which clustered together by gene expression cluster analysis, were significantly upregulated compared to WT-MARV-infected animals as early as day 2, and all three peaked on day 3 (Fig. 5 and Supplementary Fig. 7a,b). In animals infected with WT-MARV, transcript levels of these genes in the liver and spleen remained within the control range at the beginning of infection and peaked towards the end (Fig. 5 and Supplementary Fig. 7a,b). Indeed, we discerned a distinct separation in immune responses between HA-MARV and WT-MARV when MIP-1α, IP-10, and Mx2 were examined on day 3 post-infection (Supplementary Fig. 7d,e). Changes in the levels of other gene transcripts were subtler. In the livers of animals infected with HA-MARV, the pro-inflammatory cytokines TNF-α and IL-6, as well as the Th2 cytokines IL-4 and IL-5 and the Th1 cytokine IL-12, were all elevated early before decreasing over the course of infection (Fig. 5 and Supplementary Fig. 7a). In contrast, in WT-MARV-infected animals, expression of these cytokines was significantly higher at the end of infection (Fig. 5 and Supplementary Fig. 7a). In the spleens of HA-MARV-infected animals, most of these cytokines remained around the control range throughout, with the exceptions of IL-6, which peaked on day 3 and then decreased, and IL-4, which peaked on day 8 (Fig. 5 and Supplementary Fig. 7b). In WT-MARV-infected animals, there was little change in the expression of these cytokines compared to the control range (Fig. 5 and Supplementary Fig. 7b).

In the blood, innate immune responses in WT-MARV-infected hamsters peaked around day 4, at a time when innate responses were depressed in HA-MARV-infected animals (Fig. 5 and Supplementary Fig. 7c). IP-10, IL-6, TNF-α, and IL-4 transcript levels were all significantly higher in WT-MARV-infected animals on day 4 compared to HA-MARV-infected animals (Fig. 5 and Supplementary Fig. 7c). Analysis of TNF-α and IL-6 levels, alongside blood titer, once again revealed discrete immune responses in the blood of animals infected with WT- or HA-MARV (Supplementary Fig. 7f). Levels of IL-5 and IL-12 were also elevated in WT-MARV-infected animals on day 4, although the difference was not statistically significant (Fig. 5 and Supplementary Fig. 7c). Towards the terminal stage of infection, on day 6, transcript levels of some cytokines in HA-MARV-infected animals began to significantly increase, notably IL-6 and IL-4 (Fig. 5 and Supplementary Fig. 7c).

## Discussion

In this study, we have described the development and characterization of a novel Syrian golden hamster model of MHF. Although hamsters were used following the original 1967 outbreak of MARV to characterize general features of MARV pathogenesis^24^, our work here expands upon this initial work and offers a more rigorous analysis along with important and novel insights that have not been described until now. Indeed, our hamster model accurately recapitulated all the critical clinical hallmarks observed in humans and NHPs infected with MARV^6^,^8^,^10^,^22^,^23^,^25^,^26^,^27^,^28^,^29^,^30^, making it one of the most accurate small animal disease models developed for MHF to date.

Much of the pathogenesis of HA-MARV, including the coagulation abnormalities and resulting hemorrhagic manifestations, was likely driven by a severely dysregulated cytokine response, reminiscent of both a systemic inflammatory response syndrome (SIRS) and a mixed anti-inflammatory response syndrome (MARS)^31^,^32^,^33^. In general, the innate immune response elicited by HA-MARV was predominantly pro-inflammatory and possibly skewed towards a Th2 response, which is consistent with what has been observed in NHPs^6^,^8^,^10^,^23^,^34^,^35^,^36^. The patterns of cytokine/chemokine expression in both WT- and HA-MARV-infected hamsters seemed to correlate with the replication of these viruses in the primary target organs of MARV, the spleen and the liver, implying that the immune response to HA-MARV, although robust, was unable to induce a protective immune response and likely contributed to pathogenesis. The correlation between immune response and replication was not as obvious in the blood, however, where, unlike HA-MARV, WT-MARV elicited a robust, early response despite replicating to lower titers, perhaps suggesting that the early induction of a strong, systemic innate immune response is a marker of protection in MARV-infected animals. Indeed, strong, systemic inflammatory responses have been reported in presumably asymptomatic (or at least subclinical) cases of human EBOV infection^37^,^38^, another filovirus closely related to MARV. It is also worth noting that while the response elicited by HA-MARV in the blood reflects what has been observed in NHPs and other models^6^,^7^,^8^,^15^,^16^,^23^,^34^, it is not identical to the immune responses in the spleen and liver, which have typically not been characterized in other animal models, suggesting that tissue-specific innate immune signatures and their triggers are critical to understanding the key immunological factors in the pathogenesis of MHF.

Notably, although MARV encodes two proteins, VP35 and VP40, that both inhibit the IFN response^39^,^40^, HA-MARV infection nevertheless elicited an early and very strong type I IFN response in the liver and spleen. The same phenomenon was observed in MARV-infected rhesus macaques^34^, suggesting that a robust IFN response does not present a significant barrier to MARV infection. Interestingly, all three amino acid mutations acquired by HA-MARV during the course of adaptation occur in either VP35 (L33P) or VP40 (G106D and D184N) (Table 1). One of these mutations, VP40 D184N, has been previously identified in mouse-adapted (MA) Ravn virus^15^, MA-MARV variant Ci67^41^, MA-MARV variant Angola^16^, and GPA-MARV variant Musoke^42^; however, it has been shown in guinea pig cells not to affect the IFN antagonist function of VP40, but, instead, to promote nucleocapsid recruitment and virion budding^43^. Whether VP35 L33P or VP40 G106D affect the IFN antagonistic function of these proteins remains unknown, although they likely contribute to the virulence of HA-MARV in some way. It is also worth noting that the vast number of U to C mutations in the VP35 5′ non-coding region, which are likely due to adenosine deaminase activity^44^, may also contribute to VP35 protein synthesis or transcript stability, and similar mutations have been observed in the non-coding regions of other rodent adapted viruses^15^,^16^,^41^.

Intriguingly, the immune response observed in the MHF hamster model differs from that observed in the previously developed hamster model for Ebola hemorrhagic fever (EHF) using MA-EBOV^45^. Although both viruses elicited a dysregulated pro-inflammatory response, this response occurred earlier post-infection with HA-MARV and later post-infection with MA-EBOV. Likewise, a type I IFN response occurred earlier post-infection with HA-MARV and later post-infection with MA-EBOV. The delay in immune response to MA-EBOV may be explained by evidence suggesting that EBOV VP35 is more effective at inhibiting IFN induction than MARV VP35^40^,^46^; however, it is also possible that the different adaptation histories of these two viruses resulted in distinct mutations that contribute to pathogenesis in different ways. Although the hamster models of MHF and EHF share many hallmark features of filovirus hemorrhagic fever, MA-EBOV infection does not produce the characteristic petechial rash. While the differences in immune responses, among other factors, likely contribute to this discrepancy, it may be that MA-EBOV infection, which kills hamsters 3–4 days sooner than HA-MARV^45^, simply does not allow enough time for some pathogenic features, such as the rash, to develop before the animals succumb.

The development of this MHF hamster model closely recapitulating human disease will enable detailed and systematic investigations of molecular pathogenesis using the burgeoning collection of molecular research tools now available for Syrian golden hamsters. Although the historical lack of immune reagents for hamsters has resulted in a relatively poor understanding of basic hamster immunology, a number of recently developed research tools—including the hamster transcriptome and kinome^47^,^48^, microarray platforms^49^, and RT-qPCR assays to measure the host response^17^—are poised to increase the value of hamsters as an animal model. In addition, together with the EHF hamster model, the MHF hamster model will contribute to both basic and translational research for the development of medical countermeasures such as pan-filovirus therapies and bivalent vaccines.

## Methods

### Cells and Viruses

Vero E6 cells (ATCC CRL-1586) were maintained in Dulbecco’s Modified Eagle’s medium (Sigma Aldrich), supplemented with 10% heat-inactivated fetal bovine serum (ThermoFisher Scientific), 2 mM L-glutamine (ThermoFisher Scientific), and 1% Penicillin/Streptomycin (ThermoFisher Scientific) at 37 °C and 5% CO_2_. Cells were mycoplasma negative. Wild-type Marburg Virus variant Angola (WT-MARV; Marburg virus/H.sapiens-tc/AGO/2005/Angola-368) was propagated on Vero E6 cells and was used at passage 2^35^. The genome sequence for WT-MARV was determined by Sanger sequencing and was deposited in Genbank (Accession number KY047763).

### Virus Adaptation

Guinea pig-adapted (GPA) MARV was generated by inoculating Hartley guinea pigs (*Cavia porcellus*) with WT-MARV, isolating virus from the livers or spleens, and re-inoculating the passaged virus back into guinea pigs. Three passages were required to generate GPA-MARV, which was uniformly lethal. Hamster-adapted (HA) MARV (Marburg virus/RML-IRF/M.auratus-lab/AGO/2005/Angola-368-HA) was generated by inoculating Syrian golden hamsters (*Mesocricetus auratus*) with GPA-MARV, and serially passaging virus isolated from the livers and spleens back into hamsters. Five serial passages in hamsters were required to generate uniformly lethal HA-MARV used in this study. The virus originated from a liver sample from a sick hamster and was plaque purified before a larger stock was propagated on Vero E6 cells. Next generation sequencing of the virus stock using the Roche 454 Rapid XL + kit and the GSFLX 454 sequencing platform (Roche) confirmed the virus genome sequence and showed no contamination with other pathogens. A total of 78 638 reads were generated and then mapped to the reference using NEWBLER version 2.6 (Roche); 97.2% of the reads successfully mapped with a coverage of 2355X. The genome sequence for HA-MARV was deposited in Genbank (Accession numbers KY047764).

### Study Design

For the unblinded LD_50_ study, 28 male Syrian Golden hamsters (*Mesocricetus auratus*) 5 weeks of age and 80–100 g in weight were used for the determination of the dose of HA-MARV lethal in 50% of the infected animals (LD_50_). Seven groups of 4 animals were inoculated intraperitoneally (i.p.) with 10-fold serial dilutions of HA-MARV ranging from 0.001 to 1,000 PFU in 0.4 ml DMEM. All i.p. injections were given at two locations, the left and right lower abdomen. Animals were observed at least twice daily for clinical signs of disease and scored with an IACUC-approved rubric. Body weight was measured once a day, and animals were humanely euthanized when they reached the endpoint score (weight loss >20%, or the following: ataxia, lethargy (animal is reluctant to move), bloody discharge from nose, mouth, rectum or urogenital area, tachypnea, dyspnea or paralysis of the limbs). Surviving animals were kept until day 42 post inoculation; a terminal serum sample was collected and sero-conversion for MARV was confirmed by ELISA.

For the unblinded pathogenesis study, a total of 102 male hamsters 5 weeks of age and 80–100 g in weight were used. On day 0, 48 animals were i.p. inoculated with 100 LD_50_ HA-MARV in 0.4 ml DMEM (2.5 PFU/ml), and 48 animals were inoculated with an equivalent dose of WT-MARV by the same route. Six control animals received an injection of 0.4 ml DMEM alone. All i.p. injections and animal observation occurred as described above. Core body temperature was measured using IPTT-300 implantable transponders (BMDS). Beginning on day 1 post-infection and continuing until day 8, 6 HA-MARV- and 6 WT-MARV-infected animals were anesthetized via inhalational isoflurane and blood samples were collected. The animals were randomly picked from the pool. All animals were then euthanized via cardiac puncture and, following necropsy, tissue samples were collected for virological and pathological analyses. Three of the mock-infected hamsters were euthanized/necropsied on day 1 post-infection and three on day 8.

### Virus Infectivity Titration

Vero E6 cells were seeded in 48-well plates the day before titration. Tissue samples were homogenized in 1 ml DMEM and blood samples were thawed before 10-fold serial dilutions of each sample were prepared. Cells were inoculated with each diluted sample in triplicate, and virus was allowed to attach for one hour at 37 °C, after which DMEM supplemented with 2% heat-inactivated fetal bovine serum, 1% penicillin/streptomycin, and 2 mM L-glutamine was added. Cells were monitored for cytopathic effect, and 50% tissue culture infectious dose (TCID_50_) was calculated for each sample employing the Reed and Muench method^50^.

### Hematology and Coagulation Parameter Analysis

The total white blood cell count, lymphocyte, platelet and neutrophil cell counts were determined from EDTA blood with the HemaVet 950FS analyzer (Drew Scientific, Dallas, TX). Blood samples for coagulation parameter assays were collected into 1.8 mL citrate vacutainers. Plasma was separated by centrifugation and analyzed for activated partial thromboplastin time (aPTT), prothrombin time (PT), thrombin time (TT), and fibrinogen concentration on a Start4 instrument using the PTT Automate, STA Neoplastine CI plus, STA Thrombin and Fibri-Prest Automate kits, respectively (Diagnostica Stago, Parsippany, NJ).

### Pathology and Immunohistochemistry

Tissues were collected and fixed in 10% Neutral Buffered Formalin for a minimum of 7 days. Tissues were placed in cassettes and processed with a Sakura VIP-5 Tissue Tek on a 12 hour automated schedule using a graded series of ethanol, xylene, and ParaPlast Extra. Embedded tissues were sectioned at 5 μm and dried overnight at 42 °C prior to staining with hematoxylin and eosin. Specific anti-MARV immunoreactivity was detected using an anti-MARV antibody directed against the MARV nucleoprotein (FS0609 #591; kindly provided by A. Takada, Hokkaido University, Sapporo, Japan) at a 1:400 dilution. The secondary antibody was Biogenex’s anti-rabbit SS link. Activated caspase-3 was detected using an anti-active caspase-3 polyclonal antibody (G748A; Promega) at a 1:600 and the DAP Map Detection Kit (Ventana). The tissues were then processed for immunohistochemistry using Ventana Medical System’s Ultra stainer with a DABMap (Ventana Medical Systems) kit.

### Transcriptional Profiling of Host Responses by RT-qPCR

Total RNA was extracted from tissues using the RNeasy Mini Kit (Qiagen) according to manufacturer’s directions. Total RNA was extracted from whole blood using the QIAamp Viral RNA Mini Kit (Qiagen) according to manufacturer’s directions. Detection of hamster gene transcripts was performed using a reverse transcription quantitative PCR (RT-qPCR) assay essentially as described previously^17^. Briefly, all RT-qPCR reactions were performed on the Rotor-Gene 6000 thermal cycler (Qiagen) using the QuantiFast Probe RT-PCR Kit (Qiagen) according to manufactuer’s instructions. Each reaction used 50 ng of template RNA and 0.4 μM and 0.2 μM of test primers and probe, respectively (Supplementary Table 1). Reactions were multiplexed with primers and probe targeting RPL18 as the internal control, at final concentrations of 0.1 μM and 0.05 μM, respectively. All test probes were labeled with 5′ 6-FAM and 3′ BlackBerry Quencher (BBQ), whereas the control probe was labeled with 5′ Yakima Yellow and 3′ BBQ (TIB MOLBIOL). Cycling conditions were as follows: 10 min at 50 °C (initial reverse transcription) and 5 min at 95 °C (initial denaturation and polymerase activation) followed by 40 cycles of 5 seconds at 95 °C (denaturation) and 10 seconds at 60 °C (annealing and extension). Data were acquired following each cycle in the green (510 nm) and yellow (555 nm) channels. Data were analyzed using the comparative *C*_T_ method^51^,^52^, normalized to RPL18 and compared to the mock-treated hamsters.

### Statistical and Other Analysis

Data for Figs 1b–d, 2 and 3a–d, as well as Supplementary Figs 1, 4, and 5a are expressed as arithmetic mean values, plus or minus the standard error of the mean (SEM), where indicated. Data for Fig. 5 are expressed as arithmetic mean values on a log10 scale, plus or minus the SEM. Data for Supplementary Fig. 7a–c are expressed as log10 transformations of the arithmetic means and individual fold changes, respectively, in a heat map format plotted by R version 3.1.3 and R package pheatmap version 1.0.2^53^,^54^. Gene expression cluster analysis was calculated using R. Data for Supplementary Fig. 7d–f are expressed as the log10 transformations of the individual fold changes on a three-dimensional plot by R. Statistical analyses of the data were performed using the Student’s *t*-test (unpaired, parametric) and adjusted by the Benjamini-Hochberg procedure for multiple comparisons^55^. Adjusted *p*-values less than or equal to 0.05 were considered significant and marked with a single asterisk, and adjusted *p*-values less than or equal to 0.001 were considered very significant and marked with two asterisks. For histopathological analyses, tissue samples from the liver, spleen, adrenal gland, small intestine, and cervical lymph node were graded on various parameters from zero to four, with a grade of four indicating the most severe lesions. The average score for each tissue from an individual animal was calculated, and the average values for each tissue from all animals within a group were averaged. These values are displayed on line graphs in Supplementary Fig. 4.

### Biosafety and Animal Ethics Statement

All work with MARV, including infectious animal work, was performed in the biosafety level 4 facilities of the Integrated Research Facility at Rocky Mountain Laboratories (RML), National Institute of Allergy and Infectious Diseases (NIAID), National Institutes of Health (NIH). Sample inactivation and removal was performed according to standard operation protocols approved by the local institutional biosafety committee (IBC). The studies involving rodent-adapted MARV were evaluated and approved for Dual Use Research of Concern (DURC) by the RML IBC. All animal experiments were performed at RML in compliance with the guidelines of the NIAID/RML Institutional Animal Care and Use Committee (IACUC). Animal experiments were approved by the IACUC and performed following the guidelines of the Association for Assessment and Accreditation of Laboratory Animal Care, International (AAALAC) by certified staff in an AAALAC-approved facility at RML. Animal procedures were carried out under isoflurane anesthesia by trained personnel, and all efforts were made to ameliorate animal welfare and minimize suffering. Food and water were available *ad libitum* and the animals were monitored twice daily post-infection.

## Additional Information

**How to cite this article**: Marzi, A. *et al*. A hamster model for Marburg virus infection accurately recapitulates Marburg hemorrhagic fever. *Sci. Rep.* **6**, 39214; doi: 10.1038/srep39214 (2016).

**Publisher's note:** Springer Nature remains neutral with regard to jurisdictional claims in published maps and institutional affiliations.

## Supplementary Material

Supplementary Information

## Figures and Tables

**Table 1:** Genetic Changes in HA-MARV.

Nucleotide	Gene/Location	Amino Acid
Non-synonymous mutations		
U3042C	VP35	L33P
G4884A	VP40	G106D
G5117A	VP40	D184N
Synonymous mutations		
G1816A*	NP	Silent
A2847G	NP/VP35 IR	…
U4025C	VP35 5′ NCR	…
U4029C	VP35 5′ NCR	…
U4038C	VP35 5′ NCR	…
U4039C	VP35 5′ NCR	…
U4048C	VP35 5′ NCR	…
U4051C	VP35 5′ NCR	…
U4055C	VP35 5′ NCR	…
U4056C	VP35 5′ NCR	…
U4066C	VP35 5′ NCR	…
U4067C	VP35 5′ NCR	…
U4077C	VP35 5′ NCR	…
U4078C	VP35 5′ NCR	…
U4083C	VP35 5′ NCR	…
U4114C	VP35 5′ NCR	…
U4115C	VP35 5′ NCR	…
U4116C	VP35 5′ NCR	…
U4121C	VP35 5′ NCR	…
U4122C	VP35 5′ NCR	…
U4129C	VP35 5′ NCR	…
U4130C	VP35 5′ NCR	…
U4148C	VP35 5′ NCR	…
U4171C	VP35 5′ NCR	…
U4175C	VP35 5′ NCR	…
U4182C	VP35 5′ NCR	…
U4184C	VP35 5′ NCR	…
U4203C	VP35 5′ NCR	…
U4206C	VP35 5′ NCR	…
U4208C	VP35 5′ NCR	…
U4228C	VP35 5′ NCR	…
U4229C	VP35 5′ NCR	…
U4234C	VP35 5′ NCR	…
U4236C	VP35 5′ NCR	…
U4247C	VP35 5′ NCR	…
U4266C	VP35 5′ NCR	…
U4267C	VP35 5′ NCR	…
U4268C	VP35 5′ NCR	…
U4273C	VP35 5′ NCR	…
U4274C	VP35 5′ NCR	…
U4304C	VP35 5′ NCR	…
U4306C	VP35 5′ NCR	…
U4312C	VP35 5′ NCR	…
U4315C	VP35 5′ NCR	…
U4320C	VP35 5′ NCR	…
U4324C	VP35 5′ NCR	…
U4337C	VP35 5′ NCR	…
U4342C	VP35 5′ NCR	…
U4348C	VP35 5′ NCR	…
U4349C	VP35 5′ NCR	…
U4351C	VP35 5′ NCR	…
U4352C	VP35 5′ NCR	…
U4359C	VP35 5′ NCR	…
U4369C	VP35 5′ NCR	…
U4370C	VP35 5′ NCR	…
U4390C	VP35 5′ NCR	…
U5702C	VP40 5′ NCR	…
A6087G*	GP	Silent
U10127C	VP24 3′ NCR	…
A19105U*	Trailer	…

^*^Mutations likely represent polymorphisms not related to hamster adaptation, since variability at these sites was seen in two other wild-type MARV-Angola isolates (Genbank Accession Nos KR867677.1 and DQ447653.1).
