# Supplementary material for: A hamster model for Marburg virus infection accurately recapitulates Marburg hemorrhagic fever
Source: Sci Rep. 2016 Dec 15;6:39214. doi: 10.1038/srep39214 (PMC5157018; doi:10.1038/srep39214)
Supplement: Supplementary Information [file srep39214-s1.pdf]

<u>SUPPLEMENTARY INFORMATION</u>

A Hamster Model for Marburg Virus Infection Accurately Recapitulates Marburg Hemorrhagic Fever

Andrea Marzi, Logan Banadyga, Elaine Haddock, Tina Thomas, Kui Shen, Eva J. Horne,

Dana P. Scott, Heinz Feldmann, and Hideki Ebihara

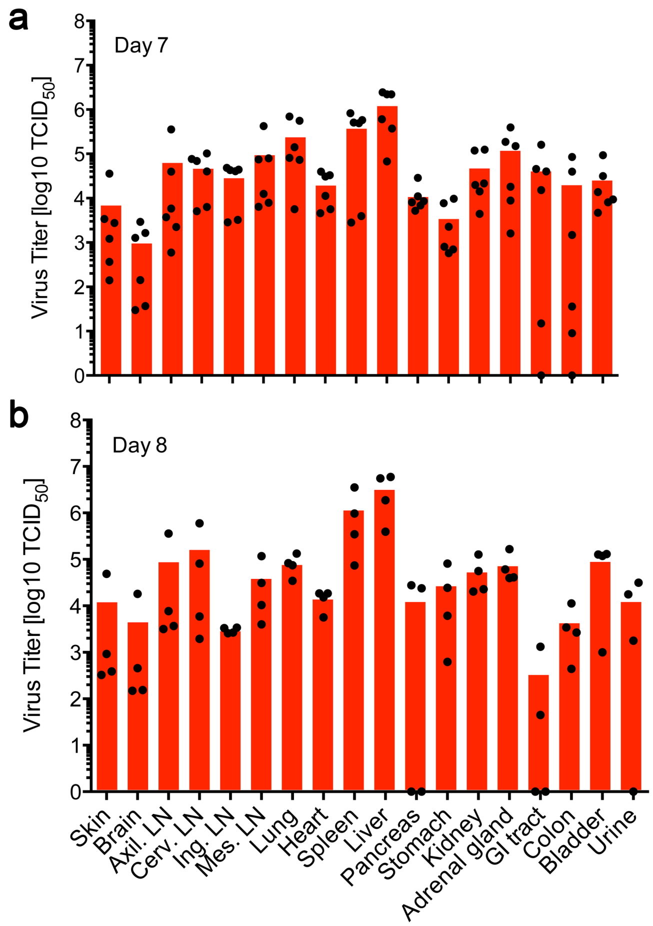

Supplementary Figure 1 | HA-MARV replicates systemically. (**a**,**b**) Virus titers, expressed as tissue culture infectious dose 50% ($TCID_{50}$) on a log10 scale, were calculated for the indicated tissues collected from HA-MARV-infected animals 7 (**a**) and 8 (**b**) days post-infection. Mean values for each time point are given as a histogram, and individual values for each hamster are indicated by black dots. n = 3-6.

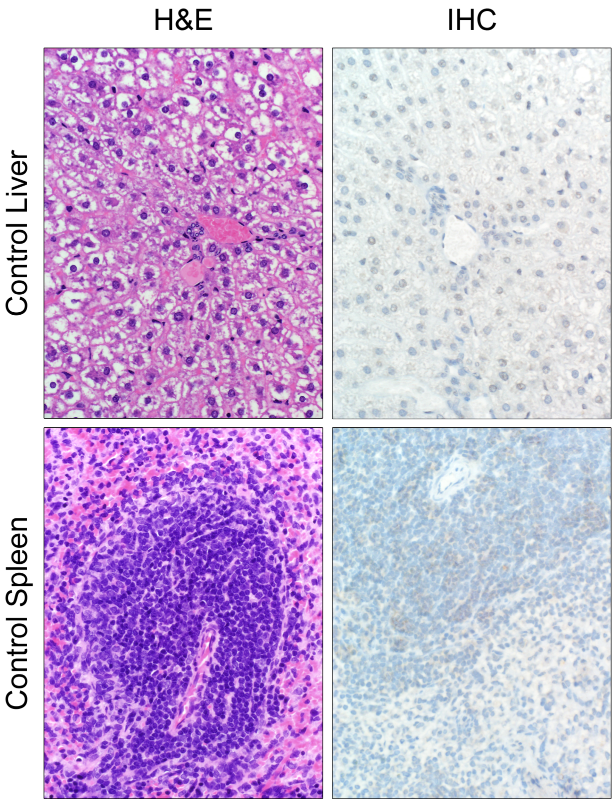

Supplementary Figure 2 | Uninfected control hamsters show no signs of pathology or virus replication. Hematoxylin and eosin (H&E) stained liver and spleen samples collected 1 day post-mock infection show no signs of pathology. Immunohistochemistry (IHC) detected no viral antigen.

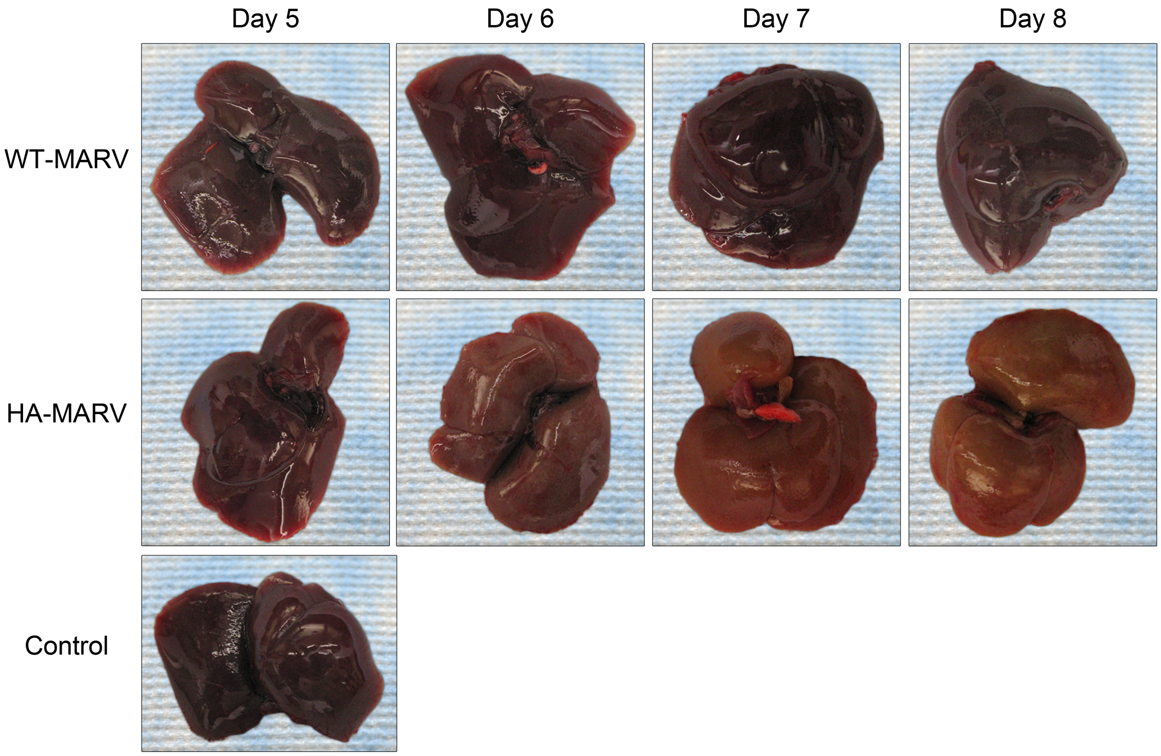

Supplementary Figure 3 | Livers from HA-MARV-infected animals show dramatic gross pathological changes. Livers collected from HA-MARV-infected animals begin to enlarge and pale in color beginning around day 5 or 6 post-infection, with gross pathology worsening over time (up to day 8). Livers collected from WT-MARV animals throughout the course of infection resembled those collected from uninfected control animals.

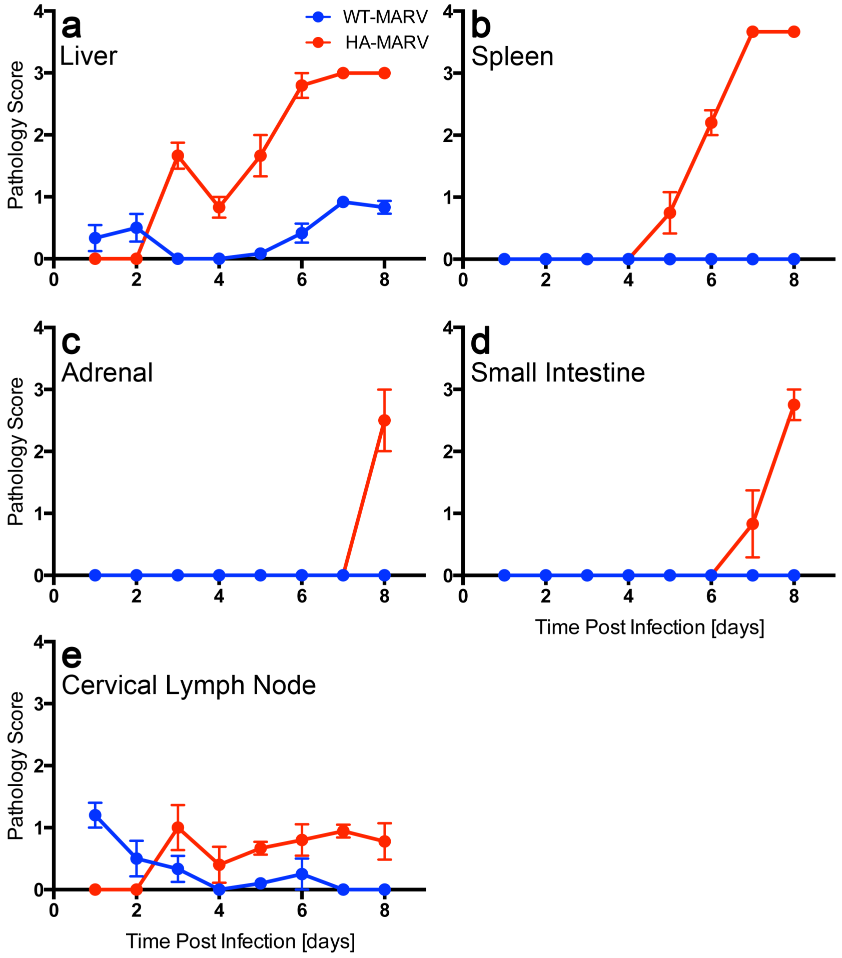

Supplementary Figure 4 | Histopathological changes are more severe in HA-MARV-infected animals. (**a-e**) Hematoxylin and eosin stained tissue samples were examined and scored on a scale of 0 to 4, with a higher number indicating more severe pathology and an increased distribution of lesions. Samples were collected from the livers (**a**), spleens (**b**), adrenal glands (**c**), small intestines (**d**), and cervical lymph nodes (**e**), and data are expressed as mean ± SEM for animals infected with WT-MARV (blue lines) and HA-MARV (red lines). n = 4-6.

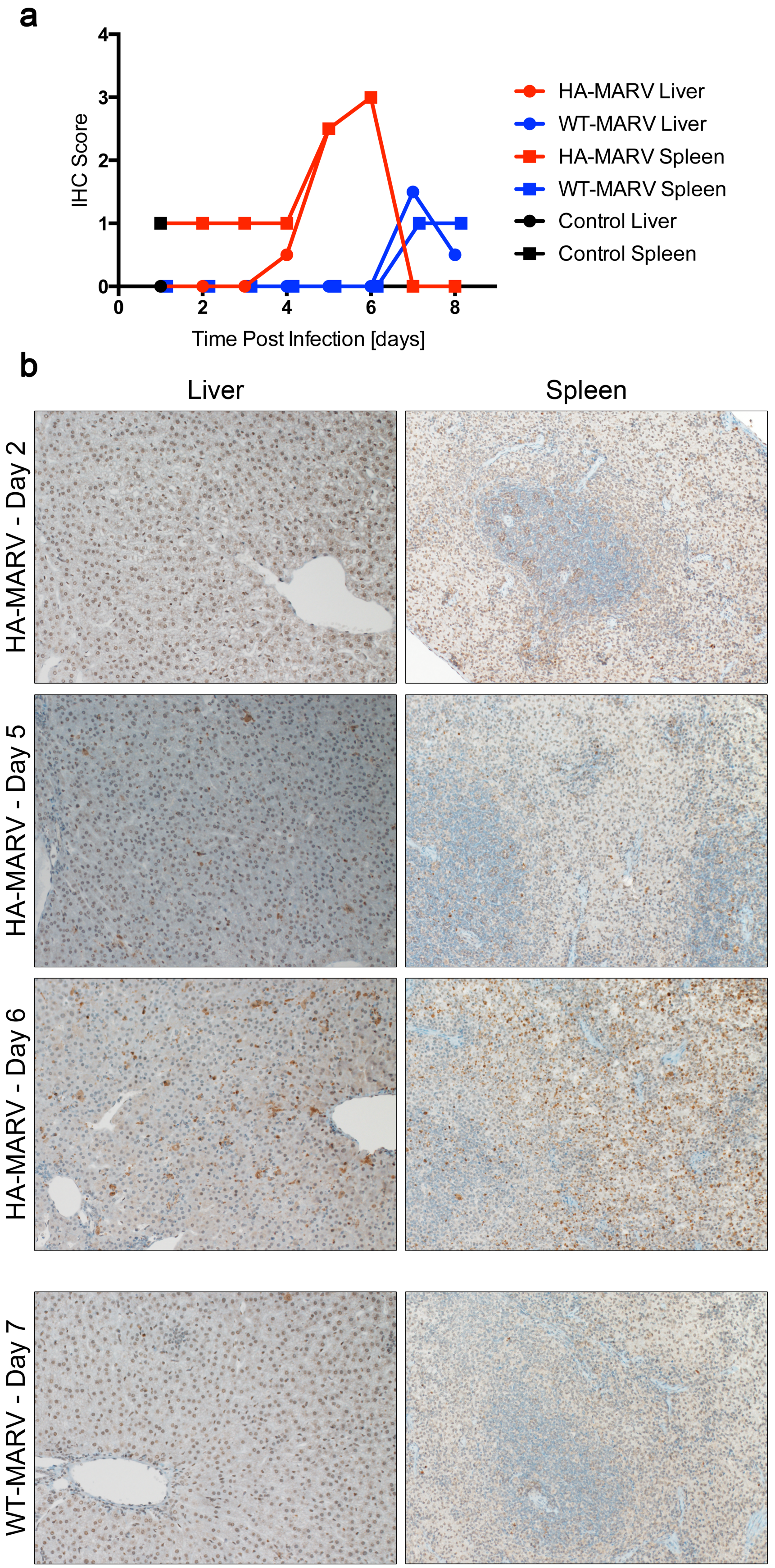
a
IHC Score
0
1
2
3
4
0
2
4
6
8
Time Post Infection [days]
HA-MARV Liver
WT-MARV Liver
HA-MARV Spleen
WT-MARV Spleen
Control Liver
Control Spleen
b
Liver
Spleen
HA-MARV - Day 2
HA-MARV - Day 5
HA-MARV - Day 6
WT-MARV - Day 7

Supplementary Figure 5 | HA-MARV activates caspase-3. (**a**,**b**) Immunohistochemistry (IHC) to detect activated caspase-3 antigen was performed on liver and spleen samples collected each day post-infection with either WT- or HA-MARV. Tissues were scored on a scale of 0 to 4, with a higher number indicating an increased distribution of antigen staining (**a**). Representative IHC images for liver and spleen samples are provided (**b**).

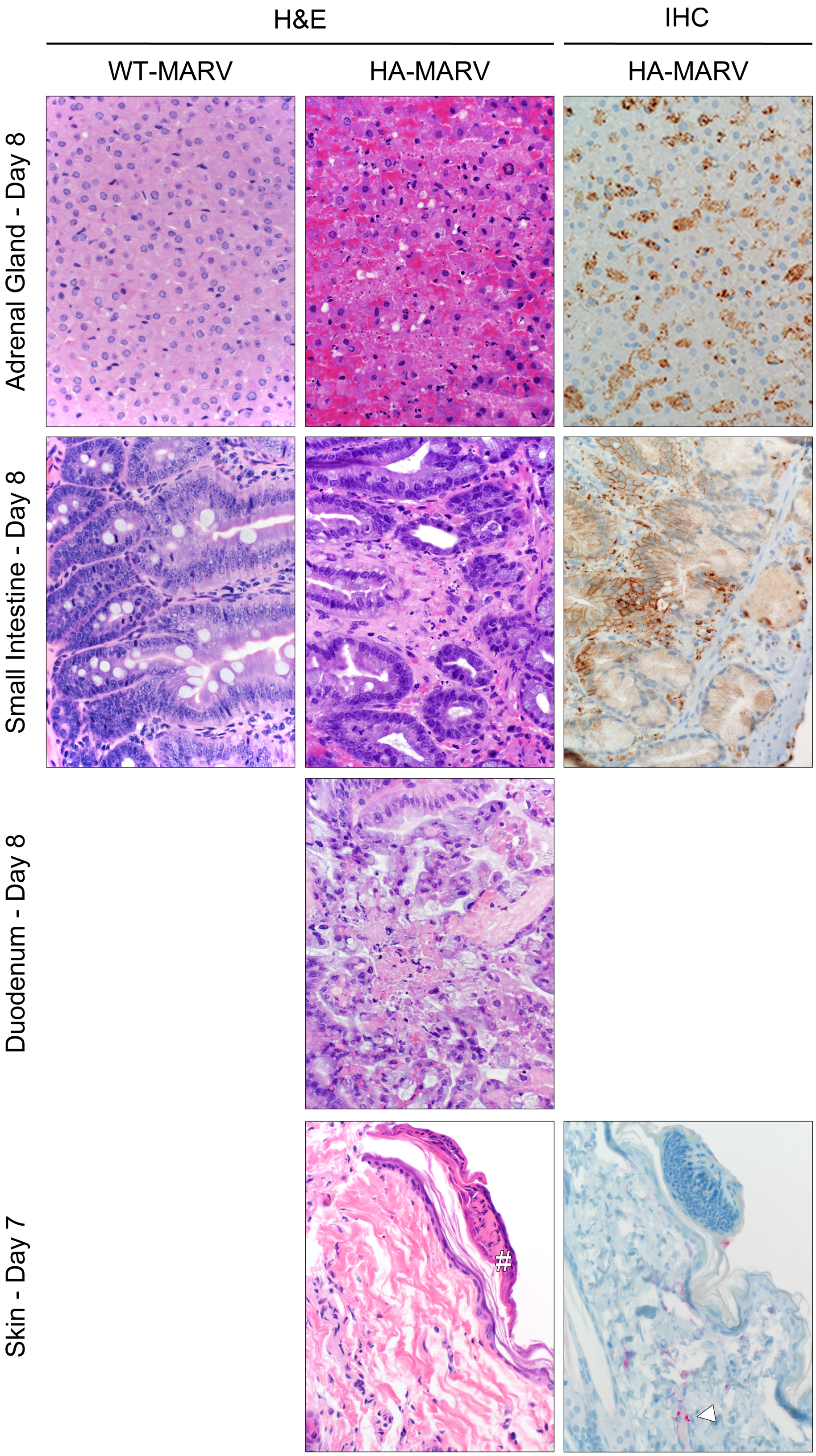
H&E
IHC
WT-MARV
HA-MARV
HA-MARV
Adrenal Gland - Day 8
Small Intestine - Day 8
Duodenum - Day 8
Skin - Day 7
#

Supplementary Figure 6 | HA-MARV-infected animals show systemic pathologic changes that correlate with virus antigen expression. Hematoxylin and eosin (H&E) stained samples collected from the adrenal glands, small intestines, and duodenum (on day 8 post-infection) or the skin (on day 7 post-infection) of animals infected with WT- or HA-MARV highlight the significant pathology caused by HA-MARV infection. Immunohistochemistry (IHC) detected MARV antigen in the adrenal gland, small intestine, and skin collected from HA-MARV-infected animals. The skin contained multifocal intracorneal pustules (#) that were adjacent to capillaries lined by MARV antigen-positive endothelial cells (white arrowhead).

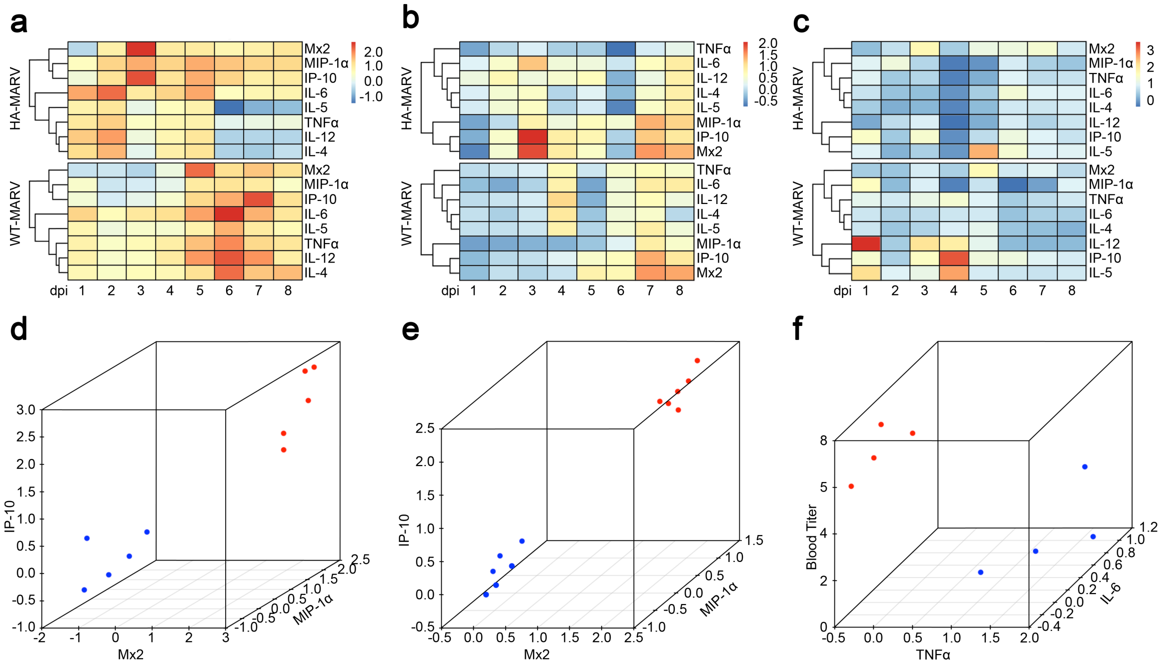

Supplementary Figure 7 | HA-MARV induces an early and strong innate immune response. (**a-c**) Heat maps depict the mean log fold change in transcript levels for the indicated genes from animals infected with HA- or WT-MARV compared to uninfected control animals. Transcript levels were quantified by qRT-PCR from samples derived from the livers (**a**), spleens (**b**), and blood (**c**). Gene expression cluster analyses are depicted to the left of each heat map. (**d-f**) 3D plots depict the log fold change in transcript level or blood titer from individual animals infected with HA-MARV (red dots) and WT-MARV (blue dots) on day 3 post-infection. Transcript levels were quantified by qRT-PCR from samples derived from the livers (**d**), spleens (**e**), and blood (**f**). n = 3-6 for the infected groups, n = 3 for the control group.

Supplementary Table 1: RT-qPCR Primer and Probe Sequences

Target	Forward Primer	Reverse Primer	Probe
**MIP1α**	GCTCTGAGCCAGGTGTCATT	GTCAGCGACGTACTCTTGGAC	6FAM-TCAGCGCAGAACTGCCGGTTT-BBQ
**IP-10**	GCCATTCATCCACAGTTGACA	CATGGTGCTGACAGTGGAGTCT	6FAM-CGTCCCGAGCCAGCCAACGA-BBQ
**IL-6**	CCTGAAAGCACTTGAAGAATTCC	GGTATGCTAAGGCACAGCACACT	6FAM-AGAAGTCACCATGAGGTCTACTCGGCAAAA-BBQ
**TNFα**	GGAGTGGCTGAGCCATCGT	AGCTGGTTGTCTTTGAGAGACATG	6FAM-CCAATGCCCTCCTGGCCAACG-BBQ
**IL-4**	CCACGGAGAAAGACCTCATCTG	GGGTCACCTCATGTTGGAAATAAA	6FAM-CAGGGCTTCCCAGGTGCTTCGCAAGT-BBQ
**IL-5**	CAAAAAGAGCAGTGTGGCC	ACCAAGGAACTCTTGCAGGT	6FAM-AACTGCCTGGCTCTCCGCCTCT-BBQ
**IL-12**	GGCCTTCCCTGGCAGAA	ATGCTGAAAGCCTGCAGTAGAAT	6FAM-CGGATCCCTACAAAGTGAAAATGAAGCTCTG-BBQ
**Mx2**	CCAGTAATGTGGACATTGCC	CATCAACGACCTTGTCTTCAGTA	6FAM-TGTCCACCAGATCAGGCTTGGTCABBQ
**RPL18**	GTTTATGAGTCGCACTAACCG	TGTTCTCTCGGCCAGGAA	YAK-TCTGTCCCTGTCCCGGATGATC-BBQ
